# Aortic Aneurysms, Chronic Kidney Disease and Metalloproteinases

**DOI:** 10.3390/biom11020194

**Published:** 2021-01-30

**Authors:** Michele Andreucci, Michele Provenzano, Teresa Faga, Ashour Michael, Gemma Patella, Pasquale Mastroroberto, Giuseppe Filiberto Serraino, Umberto Marcello Bracale, Nicola Ielapi, Raffaele Serra

**Affiliations:** 1 Department of Health Sciences, “Magna Graecia” University, I-88100 Catanzaro, Italy; andreucci@unicz.it (M.A.); teresa_faga@yahoo.it (T.F.); ashourmichael@yahoo.com (A.M.); gemmapatella@hotmail.it (G.P.); 2 Department of Medical and Surgical Sciences, University Magna Graecia of Catanzaro, Viale Europa, I-88100 Catanzaro, Italy; michiprov@hotmail.it; 3 Department of Experimental and Clinical Medicine, University of Catanzaro, I-88100 Catanzaro, Italy; mastroroberto@unicz.it (P.M.); serraino@unicz.it (G.F.S.); 4 Department of Public Health, University of Naples “Federico II”, I-80131 Naples, Italy; umbertomarcello.bracale@unina.it; 5 Department of Public Health and Infectious Disease, “Sapienza” University of Rome, I-00185 Roma, Italy; nicola.ielapi@uniroma1.it; 6 Interuniversity Center of Phlebolymphology (CIFL), “Magna Graecia” University, I-88100 Catanzaro, Italy

**Keywords:** MPs, proteinuria, eGFR, aneurysm expansion, extracellular matrix, cardiovascular risk, end-stage kidney disease, renal tubular injury

## Abstract

Metalloproteinases (MPs) are proteolytic enzymes involved in extracellular matrix deposition, regulation of cellular signals of inflammation, proliferation, and apoptosis. Metalloproteinases are classified into three families: Matrix-MPs (MMPs), A-Disintegrin-and-Metalloprotease (ADAMs), and the A-Disintegrin-and-Metalloproteinase-with-Thrombospondin-1-like-Domains (ADAMTS). Previous studies showed that MPs are involved in the development of aortic aneurysms (AA) and, concomitantly, in the onset of chronic kidney disease (CKD). CKD has been, per se, associated with an increased risk for AA. The aim of this review is to examine the pathways that may associate MPs with CKD and AA. Several MMPs, such as MMP-2, -8, -9, and TIMP-1 have been shown to damage the AA wall and to have a toxic effect on renal tubular cells, leading to fibrosis. Similarly, ADAM10 and 17 have been shown to degrade collagen in the AA wall and to worsen kidney function via pro-inflammatory stimuli, the impairment of the Renin-Angiotensin-Aldosterone System, and the degradation of structural proteins. Moreover, MMP-2 and -9 inhibitors reduced aneurysm growth and albuminuria in experimental and human studies. It would be important, in the future, to expand research on MPs from both a prognostic, namely, to refine risk stratification in CKD patients, and a predictive perspective, likely to improve prognosis in response to targeted treatments.

## 1. Introduction

Aortic aneurysms (AA) are defined as segmental, full-thickness dilations of the aorta resulting in a 50% increase in diameter, when compared with the normal aortic diameter [1]. The natural history of aneurysm is characterized by a slow expansion with a concomitant raised risk of rupture. The rate of aneurysm enlargement depends on several factors, including its etiology and location, being more frequent in the abdominal than in thoracic aorta [2]. Rupture is the most severe and most feared complication of larger aneurysms. In patients already diagnosed with thoracic AA, the rate of rupture ranged from about 30 to 70% with a mortality risk of 50% [3]. The mortality risk, after abdominal AA rupture, was even higher and reported in up to 85% of cases [2,4]. The importance of such findings is reinforced by the fact that the incidence of AA rupture and mortality are increasing [5]. According to the Global Burden of Disease, mortality due to aortic aneurysms (encompassing all tracts of the aorta) increased by 23.7% in the time period 2007–2017 [6]. Therefore, these alarming data suggest the importance of improving prevention and therapeutic strategies for high-risk patients. If the accurate screenings of AA have been shown to yield lower mortality rates, the randomized trials, evaluating the efficacy of operative strategies, have failed in their prognostic benefits [7]. However, finding a pharmacological treatment to slow the enlargement and progression of AA poses a major challenge. Many intervention studies, testing the effect of statins and angiotensin-converting enzyme inhibitors have failed the test, to the extent that an Editorial, recently published in the Annals of Internal Medicine Journal, was provocatively titled “Still no Pill”, discussing this point [8]. A population at increased risk for the development of AAs and their complications is represented by chronic kidney disease (CKD) patients [9]. Chronic Kidney Disease is defined as the presence, for at least 3 months, of abnormalities across the urinary system and/or out-of-range levels of two kidney measures, namely, estimated Glomerular Filtration Rate (eGFR) and proteinuria [10]. It has been shown that both low eGFR and high proteinuria levels forecast cardiovascular events, including peripheral vascular disease [11,12]. More specifically, both the two kidney measures, eGFR and proteinuria, have been independently associated with an increased risk for incident abdominal AA as well as with an increase in diameter of the abdominal aorta [9]. A key pathogenetic factor, which is common to both these conditions, is the imbalance of the extracellular matrix (ECM) [13]. In this specific context, a pivotal role is exerted by metalloproteinases (MPs). Metalloproteinases are zinc-containing enzymes, which belong to the big family of metzincin proteases [14]. This metzincin family encompasses three large groups of proteolytic enzymes with peculiar characteristics: matrix metalloproteinases (MMPs), the A Disintegrin and Metalloproteinase (ADAMs) family and the A Disintegrin and Metalloproteinase with Thrombospondin-1-like Domains (ADAMTS) [15]. Several studies have shown that MPs participate in several steps of the development of AA [15,16]. Increased circulating levels of neutrophil gelatinase-associated lipocalin (NGAL) and MMP-9 have been documented in patients with AA [17,18]. Similarly, overexpression of several ADAMs and ADAMTS promotes AA development and expansion [15]. Interestingly, MPs are concomitantly involved both in vascular and renal damage through similar pathogenetic pathways. Indeed, increased levels of MMP-2 and MMP-9 and ADAM-17 promote inflammation, atrophy and fibrosis of renal tissue, thus accelerating kidney damage in CKD patients [13,19]. The aim of this review article is to inspect the role of MPs in the development of AA, their link with CKD and the mechanisms by which each of these conditions impairs the prognosis of the other.

## 2. Materials and Methods

We used two databases, namely, PubMed and ISI Web of science databases for literary research by including the following terms: “CKD”, “chronic kidney disease”, “metalloproteinases”, “MP”, “MMP”, “chronic kidney disease AND aortic aneurysms” “aortic aneurysms AND metalloproteinases” “thoracic aortic aneurysms AND chronic kidney disease”. Titles and abstracts were screened by three authors (Raffaele Serra, Michele Andreucci, and Michele Provenzano) to identify potentially relevant studies. All potentially eligible studies were subsequently evaluated in detail by one reviewer and three authors (Michele Provenzano, Michele Andreucci, Nicola Ielapi, and Raffaele Serra) through consideration of the full text. We also evaluated the reference lists of retrieved articles for potential relevant publications. Clinical trial, meta-analyses, narrative review, and systematic reviews published in the last 15 years were included. The bibliographies of all relevant articles were manually screened to identify additional studies. Studies were not included if they were in a language other than English, and if they did not fit the research question. 

## 3. Results

### 3.1. Study Selection

The first database searches provided 215 studies from PubMed and 450 from ISI Web of Science published within the last 15 years. After the evaluation of titles, abstracts, and bibliographies of the relevant articles, we evaluated 95 eligible full text articles. The current evidence around the role of MPs in the pathogenesis of aortic aneurysms, the association between MPs and CKD, and the patterns shared between MPs and aortic aneurysms in the specific condition of CKD, are described below. 

### 3.2. Association between Metalloproteinases and Aortic Aneurysms 

Herein, we present the role of each MP family in the pathogenesis of aortic aneurysms. Matrix metalloproteinases are synthetized as non-functioning proenzymes, which are then activated by intracellular convertases or extracellular peptidases, the latter also including other MMPs [20,21]. It has been shown that MMPs can exert their proteolytic function on a wide range of structures, such as ECM components (proteoglycans, collagens, and gelatins) and cell membrane receptors, and are involved in the signal regulation of several chemokines, cytokines, and growth factors [22]. As a result of these multiple interactions, MMPs intervene in cell-to-cell and cell-to-ECM adhesion, cell survival and proliferation. All these functions share two important characteristics: they are crucial to cell and organ homeostasis and they are generalized systemic functions. In addition to this general definition, MMPs are more specifically classified into 6 groups, based on their structure, as follows: collagenases, gelatinases, stromelysins, matrilysins, membrane-type MMPs, and other MMPs [13]. The collagenases are so called because they cleave collagen in a synergic action with gelatinases and are mainly represented by MMP-1, -8, -13 [19]. Gelatinases (MMP-2 and -9) play different functions: they cleave ECM components, such as aggrecan, elastin, and collagens [23]; in addition, they are involved in the regulation of several cytokines, such as transforming growth factor-beta1 (TGF-β1), tumor necrosis factor-alpha (TNF-α), and chemokines, e.g., IL-8 [24]. Stromelysins degrade many substrates, namely, fibronectin, laminin, and proteoglycans, but not type I collagen [25]. MMP-3, -10, and -11 belong to the stromelysins family. Matrilysins (MMP-7 and -26) are characterized by a smaller molecular size, compared with other MMPs. They act by degrading several ECM components, such as fibronectin, entactin, and elastin, and modulate the effect of various cytokines [26]. Membrane-type MMPs are transmembrane (MMP-14, -15, -16, and -24) or membrane-anchored (MMP-17 and -25) proteins, which have proteolytic effects on the extracellular environment structures and also have the ability to activate other MMPs [27]. A further class of MMPs, generically defined as “other MMPs”, encompasses enzymes that differ from the typical MMPs because of two main reasons: firstly, these other MMPs are tissue or cell specific, and secondly, they are synthetized under specific stimuli [28]. The activities of the MMPs are regulated by endogenous inhibitors of MMPs (tissue inhibitors of metalloproteinases, TIMPs), which bind the N-terminal domain in the catalytic site of MMPs leading to inactivation. It has been demonstrated that MMPs play a crucial role in the pathogenesis of aneurysmal vascular disease. In fact, the vascular remodeling and alterations of ECM components are directly involved in the structural and functional changes in the vessel wall that finally result in vessel dilation [29]. An increased proteolytic activity on collagen and elastin in the context of aneurysm walls has been described in previous studies. With respect to abdominal aortic aneurysms, the gelatinases MMP-2 and MMP-9 have been found to be overexpressed in the aneurysm wall [30]. MMPs are centrally involved in the inflammatory and non-inflammatory processes that start the aneurysm formation. In fact, during aneurysm formation, there is an increased number of macrophages secreting MMP-2 and -9, which are mainly located at the adventitial–medial junction [31]. Other than macrophages, MMPs are also synthetized by smooth muscle cells, which are present in the aneurysm wall [32]. Moreover, through a fascinating analysis conducted in both ruptured and unruptured abdominal aortic biopsies, Wilson and colleagues showed that MMP-8 and -9 are particularly increased at the site of aortic rupture [33]. This led them to hypothesize an effective role of MMP-8, a potent collagenase degrading type I collagen, which is expressed in greater concentration compared with type III collagen in the human aortic wall [34]. Hence, the localization of MMPs across the aortic wall promotes the weakening of the wall. However, the presence of hemodynamic forces contributes to aneurysm rupture. The amount of MMP-9 was found to be increased in abdominal aortic aneurysm (AAA) of small–medium size (up to 6.9 cm) but not in larger aneurysms [8]. Further, the degree of local activity of MMPs was not different between ruptured and unruptured aneurysms, thus suggesting that the final degradation of the matrix is, at least in part, dependent on hemodynamic factors [33]. Several studies have investigated the role of MMPs and TIMPs in the context of ascending and thoracic aorta aneurysms (TAA) [35,36]. In patients with aneurysms of TAA, increased circulating levels of MMP-1, -2 and TIMP-1 were reported and were also associated with the shear stress which, in turn, promotes aortic structure remodeling and consequently favors aneurysm expansion [35]. Similarly, circulating levels of MMP-2 and MMP-8 have been shown to modulate the elasticity of aortic wall in patients TAA [36]. Several other studies evaluated the association between the neutrophil gelatinase-associated lipocalin (NGAL) and aortic aneurysms [17,37]. The rationale of this hypothesis is that NGAL interacts with MMP-9, preventing its degradation [13]. In patients with AA, NGAL is released by the polymorphonuclear neutrophils present in the luminal layer of the AA thrombus. Intriguingly, it has been shown that circulating levels of NGAL are higher in patients with AA compared with healthy control subjects and even the tissue expression of this marker is higher in the aneurysm wall than in the healthy endothelium wall of the same patient [17,37]. ADAMs are a family of transmembrane and secreted endopeptidases [15]. They consist of multiple domains, namely, a pro-domain, a metalloprotease domain, a disintegrin domain, a cysteine-rich domain, an epidermal-growth factor-like domain, and a transmembrane domain. ADAMs are classified in several groups, from ADAM1 to ADAM33. Importantly, in patients undergoing surgical repair for AA, high levels of activated ADAM10 and ADAM17 were found in the abluminal layer of the intraluminal aneurysm thrombus [38]. This concept seemed to be particularly important since the abluminal layer represents the oldest stratum of the thrombus, and it is more adjacent to the aortic wall. The activated ADAMs are able to directly degrade structural elements of the arterial wall and to activate other MMPs and the TNF-α/c-JNK pathway, which, as part of a vicious circuit, contribute to damaging the aneurysm [15,38]. The ADAMTS family encompasses 19 members of multidomain extracellular proteases and, similarly to MMPs, they are synthetized as inactive pro-enzymes that are subsequently activated [39]. ADAMTS 1 and -4 have been associated with an increased degree of versican degradation in the aortic wall and in AA, with a consequent reduction in neo-intima thickening and a rise in pro-inflammatory alterations [40].

### 3.3. Chronic Kidney Disease and Increased Risk for Aortic Aneurysms

Chronic Kidney Disease is a clinical condition characterized by a poor prognosis [41]. Patients with CKD are prone to developing several clinical endpoints, which are usually classified as renal and cardiovascular endpoints [42]. If the renal endpoints are mainly represented by eGFR decline over time and end-stage kidney disease (ESKD, the terminal phase of CKD which requires substitutive therapies, such as dialysis), the CV endpoints encompass several fatal and non-fatal events, such as myocardial infarction, stroke, heart failure, CV mortality, and peripheral vascular disease. The incidence rate of CV fatal and non-fatal outcomes in CKD patients overcomes the rates of renal outcome, this being true even in CKD patients followed by a Nephrologist (rather than by General Practitioners), who are patients at extremely significant renal risk [43,44]. In a historical cohort of patients with diabetic CKD followed by nephrologists, Minutolo et al. found that the incidence rate for CV events was 6.55 per 100 patients/year, slightly higher than that observed for the incidence of ESKD in the same subgroup (6.04 per 100 patients/year) [42]. Hence, the need to improve the CV prognosis of CKD patients has become urgent for both researchers and clinicians. The available research findings can allow us to assert that the presence of CKD increases the risk for AA [9,45]. An analysis of United States Veterans, aged 65–75 years at increased CV risk, showed that subjects with impaired kidney function, namely, with eGFR<60 mL/min/1.73 m^2^, had a significantly higher frequency of AA (37%) than those with eGFR above 60 mL/min/1.73 m^2^ (24%). This scenario was also confirmed when the association between eGFR and AA was adjusted for the other CV risk factors, with an 81% increased risk for AA still being present in the lower eGFR category [45]. Accordingly, in a Pittsburg subgroup of the Cardiovascular Health Study (CHS), which included subjects of 65 years old or older who underwent screening for AA, the presence of aneurysms was significantly associated with numerous cardiovascular risk factors, such as smoking habit, atherosclerotic diseases, hypercholesterolemia, and, as a renal parameter, the increase in serum creatinine [46]. More recently, Matsushita et al. provided more comprehensive evidence regarding the association between both kidney measures, eGFR and albuminuria and AA [9]. In this study, which consisted of an observational analysis including more than 10,000 patients of the Atherosclerosis Risk in Communities (ARIC) Study, the risk for incident AA increased by 4.4-fold for eGFR below 30 mL/min and about 2.5-fold for the albuminuria stratum ≥ 300 mg/g. The link between CKD and AA was also investigated from a mechanical point of view. CKD has been recognized as an independent risk factor for AA rupture [47]. In fact, the AA wall in patients with CKD was characterized by low thickness and low failure tension, two features that are likely due to the decreased content in collagen fibers, specifically in this patient population [48].

### 3.4. Role of Metalloproteinases in Chronic Kidney Disease Progression

**Figure 1:**
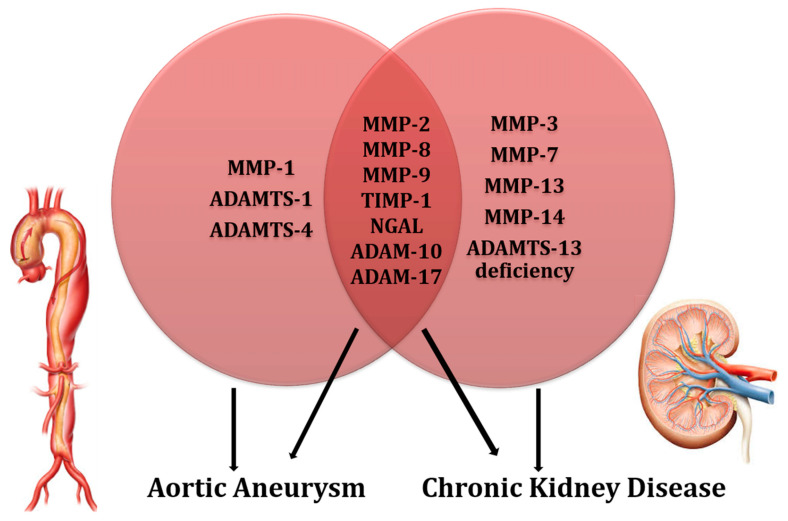
Metalloproteinases (MPs) in the pathogenesis of CKD and AA. The two circles contain the MPs associated with each clinical condition (indicated with the arrow), whereas the intersection area (darker colored) shows the MPs associated with both conditions.

Owing to their wide expression and functions, MMPs have been investigated in the field of Chronic Kidney Disease. First of all, circulating and urinary levels of MMPs have been measured in CKD patients, and these levels have been correlated with other typical signs of kidney damage [49,50,51,52,53]. In patients with diabetic nephropathy, which represents the leading cause of CKD progression to date, increased blood and urinary levels of MMP-2, -7, -8, -9, and NGAL have been reported [49,50,51,54]. Among them, MMP-9 levels were directly correlated with the degree of proteinuria, the main sign of kidney damage, which accelerates the progression of CKD toward ESKD and significantly increases the CV risk as well. Other than in diabetic CKD, similar patterns have been observed in disparate kidney diseases. This is particularly true for glomerular kidney diseases. In fact, expression of MMP-2 and -9 was found to be altered in focal segmental glomerulosclerosis, membranous nephropathy, and vasculitis [52,53]. All these aetiologies of kidney disease, albeit being dissimilar and with different degrees of CKD progression, share at their basis the alteration in renal ECM and inflammation, which are processes that lead to renal fibrosis over time. MMP-2, -7, -9, and TIMP-1 promote inflammation and imbalances in the extracellular matrix [55,56]. In particular, the gelatinases MMP-2 and -9 have a direct effect on renal tubular cells, especially by impairing the cell-to-cell adhesion, a mechanism that also involves the E-cadherin protein [57]. This process results in the loss of cellular junctions and alterations of tubular basement membrane. Altogether, these mechanisms lead to epithelial-to-mesenchymal transition (EMT), a pathway through which epithelial tubular cells differentiate to mesenchymal cells, which in turn contribute to renal fibrosis over time. Accordingly, Cheng and colleagues showed that MMP-2 overexpression in the kidney is sufficient to generate all the phases of renal fibrosis via pathways involving TGF-β signaling, EMT, and collagen type IV degradation [57]. The importance of such findings is crucial when considering that both MMP-2 and -9 are produced intrinsically in renal tubular cells [58]. Beyond the gelatinases, other MMPs play a role in determining kidney damage. MMP-3, -13, and -14, which belong to the stromelysins, collagenases, and membrane-type MMPs, respectively, have been found to be overexpressed in kidney fibrosis [59]. Another clinically relevant consequence of the deleterious actions of MMPs across the kidney is their association with CKD progression. CKD is classified into several stages based on eGFR and albuminuria levels, according to current KDIGO guidelines [60]. Matrix Metalloproteinase expression was found to progressively increase when moving from Stage I to V of CKD, thus testifying that they may per se accelerate kidney damage, with a parallel raising of CV, other than renal risk [58]. Several ADAMs have been implicated in the pathogenesis of atherosclerosis, malignancies, and other diseases [61]. Among them, two enzymes, namely, ADAM10 and -17, have been shown to be strictly associated with CKD [62]. ADAM17 participates at least in three important mechanisms of renal damage. It cleaves the Angiotensin Converting Enzyme-2 (ACE2), whose physiological function is to counterbalance the accumulation of Angiotensin-II in renal tissue [63]. Hence, Renal-Angiotensin-Aldosterone System (RAAS) regulation, which is crucial for renal function impairment, is altered. Moreover, the increased concentration of urinary ACE2 has been proposed as a biomarker for the early detection of diabetic kidney disease, a high CV and renal risk condition [62]. ADAM17 also degrades endothelial protein C receptor, thus reducing the anti-inflammatory properties of protein C on systemic and renal (i.e., glomeruli) vasculature [64]. Further, ADAM17 acts via the TNF-α/EGFR pathway leading to pro-inflammatory and pro-fibrotic stimuli, particularly by enhancing inflammatory cell migration in the kidney [65]. These mechanisms have been demonstrated in various chronic kidney conditions, such as lupus nephritis, polycystic kidney disease (PKD), diabetic nephropathy, and kidney allograft rejection [62]. The shedding of Cell adhesion molecule 1 (CADM1) mediated by ADAM10, has been associated with both enhanced apoptosis of renal tubular cells and increased serum creatinine levels in humans with renal atherosclerotic and diabetic diseases [66]. Similarly, ADAM10 also degrades E-cadherin; this has been shown to impair renal damage in Autosomal Dominant PKD patients [67]. Both ADAM10 and -17 act by degrading the adhesion molecule CXCL16, expressed in tubular renal cells and also in podocytes. Increased urinary levels of CXCL16 have been observed in patients with acute tubular necrosis and lupus nephritis [68]. Deficiency of ADAMTS13 is the causative factor of most cases of thrombotic thrombocytopaenic purpura, since this MP is crucial in von Willebrand factor multimers to avoid platelet activation [69]. In some cases, ADAMTS13 deficiency has been associated with renal damage in both glomerular and tubular damage [70]. A summary of the mechanisms involving MPs in AA and CKD patients is described in Table 1, whereas the MPs that are shared in both these conditions are shown in Figure 1.

## 4. Discussion

**Figure 2:**
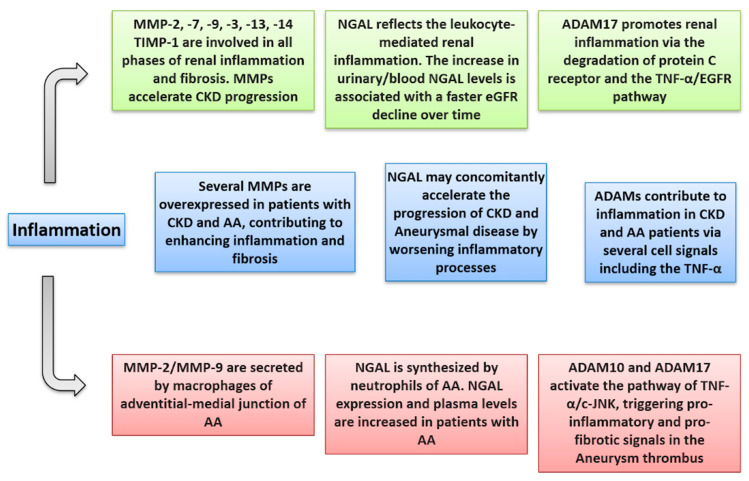
Inflammatory alterations that metalloproteinases may exert in CKD (green), aortic aneurysm (red), and both conditions (light blue).

**Figure 3:**
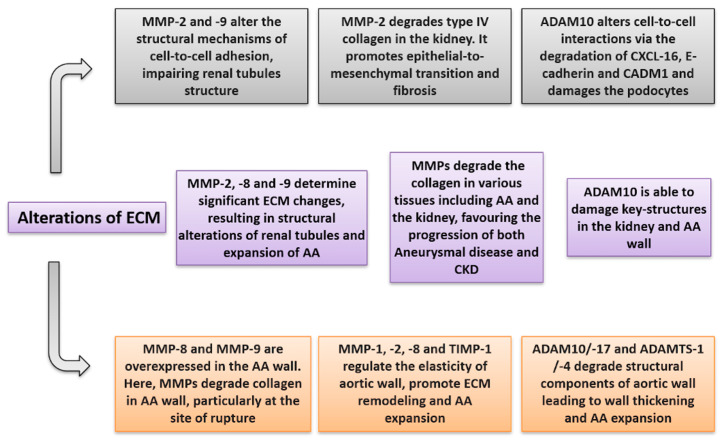
Alterations of extracellular matrix (ECM) caused by metalloproteinases in CKD (grey), aortic aneurysm (orange), and both conditions (violet).

Given the crucial pathogenetic role of MPs in triggering vascular and renal damage, a large number of studies, to date, have been carried out to better understand their potential prognostic and predictive role in a wide spectrum of CV disease and in CKD as well [20,71]. Within the wide spectrum of CV diseases, many experimental and human studies have clarified that MPs play a major role in the development, enlargement, and rupture of AA [29,30,31,32]. On the other hand, imbalances in blood and urine levels of MPs have been associated with the renal glomerular and tubular damage underlying virtually all causes of CKD, such as diabetic nephropathy, glomerulonephritis, atherosclerotic nephropathy, and Autosomal Dominant PKD [49,50,51,52,61,62]. These findings are of particular interest since CKD is per se associated with an extremely high risk for CV diseases, including AA. It has been recently speculated that one of the reasons for which impaired kidney function, evidenced by low eGFR and high albuminuria levels, is attributable to the dual role of MPs implicated in the pathogenesis of both renal injury and aneurysms [9]. This notwithstanding, this is the first review article depicting, as its primary aim, the potential intercurrent relationships between these three entities, namely, CKD, AA, and MPs. There are several reasons which may allow us to hypothesize that MPs may impair the risk for AA in CKD patients. First of all, among the large number of MPs, it is possible to recognize a “cluster” of MPs, within each group, that are concomitantly involved in both aneurysm and renal damage impairment. Between MMPs, MMP-2, -8, -9, and TIMP-1 are overexpressed and hyperactivated in the aneurysm wall [30,31,32,33,34,35,36]. At the same time, these MMPs have been demonstrated to be able to both damage tubular cells and to activate a series of pro-inflammatory and pro-fibrotic signals in the kidney that ultimately result in accelerating CKD progression [57]. Circulating levels of NGAL, with a consequent enhanced effect of MMP-9, are increased in patients with both AA and CKD, with those already in this latter condition being associated with a faster eGFR decline [17,37,72]. Similarities are also detected regarding the ADAMs family. The ADAM-10 and -17 have been shown to degrade the arterial wall, where they are mainly located, and at the same time, both these MPs are able to damage the kidney by altering RAAS regulation, reducing anti-inflammatory stimuli, as well as by degrading several cell-to-cell adhesion molecules [62]. The complex mechanisms by which MPs impair CKD and aneurysmal disease are depicted in Figure 2 (inflammatory mechanisms) and Figure 3 (alterations of the extracellular matrix).

Second, CKD is a condition associated with endothelial and, more generically, systemic vascular alterations, regardless of any other CV risk factors, such as diabetes, dyslipidemia, or smoking habit [12]. Arteries of CKD patients frequently present with atherosclerosis and increased stiffness, compared with those of healthy subjects [73,74]. Furthermore, the aneurysm wall of CKD patients is particularly poor in collagen content, and it is also particularly thick, with MPs being strictly associated with all these features [48]. Hence, it is conceivable that the risk of developing a severe aneurysmatic condition is also increased in CKD patients because of the latent vascular impairment, whose development is triggered by MPs. Another important element that needs to be mentioned is that both CKD patients and patients with AA generally present with a high prevalence of history of previous CV disease [42,46]. Prevalence of an anamnestic history of CV disease (stroke, myocardial infarction, peripheral vascular disease, and heart failure) reached 34.4% of a population of CKD patients referred to nephrologists [75]. Such a high CV risk may represent the ideal environment for MPs to exercise their harmful functions. Owing to these findings, we generate the hypothesis that MMPs may explain, at least in part, the increased CV risk (including that of AA) in CKD patients. The true knowledge of CV risk in CKD patients is still incomplete. Tonelli and colleagues, in a large cohort analysis including more than 1 million subjects of the Alberta Kidney Disease Network, showed that CV event rate in CKD patients is even higher than in patients with type II diabetes, and argued that CKD may be considered a CV risk equivalent [76]. Considering CKD as a risk equivalent has generated many debates, because it would be translated into clinical practice in an aggressive pharmacological treatment (e.g., extensive use of statins) to all patients with CKD irrespective of age, gender, eGFR, albuminuria levels, or renal diagnoses. Conversely, a more careful approach to CV risk in CKD has been prompted by the International Society of Nephrology, and consists of improving the knowledge of biomarkers that would allow to better forecast CV events [77]. Metalloproteinases may be the true candidate for this purpose. Further studies evaluating the association between MPs and all the major CV endpoints, including AA, in the particular setting of CKD patients referred to the tertiary care setting are needed. Moreover, in addition to a prognostic role, MPs may represent a valid therapeutic target for high CV risk patients, such as CKD patients. Pharmacological agents that interfere with MP pathways have been tested in preclinical and in first clinical studies in patients with CKD and in patients with CV disease [13,71]. The antibiotic doxycycline inhibits the activity of MMP-2 and -9 and has been shown to reduce proteinuria in patients with diabetic nephropathy [78,79]. Similar effects are reached by the novel MMP inhibitors, BB-1101and BB-94 [80]. However, the proteinuria (or albuminuria) reduction, albeit being associated with a reduction in CV risk and with a slower decline in eGFR, is not used as a hard endpoint of clinical trials yet [81]. In aneurysmal disease, the pharmacological inhibition of MP activity was tested in experimental and in vivo studies [71]. Once again, doxycycline significantly reduced MMP-9 levels in humans and delayed aneurysm expansion in animal models by a mechanism consisting of strengthening the aortic wall through collagen deposition [82,83]. In both cases of the CV and renal disease settings, it seems desirable that more robust and large clinical trials testing the hypothesis of a consistent reduction in AA expansion and CV risk in CKD patients should be started in the near future. Such a strategy would be important to enlarge the therapeutic armamentarium for patients with CKD. Clinical research has led to the recent introduction of novel agents for CKD patients. These include the sodium-glucose-cotransporter 2 inhibitors (SGLT2i), the selective endothelin A receptor antagonists, and the novel non-steroidal mineralocorticoid receptor antagonists. These treatments have been shown, when taken individually, to be effective in lowering both CV and renal risk in CKD [84]. How to combine these treatments to obtain a true balanced association that is effective and safe for each individual remains unclear. MP inhibitors could enter into these precision medicine strategies. This is true also because these drugs could potentiate the effect of SGLT2i via the reduction in epithelial-to-mesenchymal transition and systemic fibrosis [13]. In general, clinical research is oriented toward a stronger development and implementation of novel biomarkers that may improve diagnosis, prognosis, and treatment of multifactorial and complex diseases in the future [85].

## 5. Conclusions

In conclusion, MPs are centrally implicated in the pathophysiological mechanism at the bases of CKD and AA. Furthermore, CKD increases the risk for AA. These two clinical conditions, CKD and AA, should be considered together in clinical and research settings. MPs may play a relevant prognostic role, by improving risk stratification of high-risk patients, and a predictive role, since their therapeutic inhibition may slow the risk of both CKD progression and AA expansion. 

## Figures and Tables

**Table 1:** Summary of the association between metalloproteinases (MPs), chronic kidney disease, and aortic aneurysm.

	Key-Concepts
**MPs and Aortic Aneurysm**	• MMP-2 and MMP-9 are activated and overexpressed in the Aortic Aneurysm wall. Moreover, inflammatory cells producing MMP-2 and -9 are increased in the adventitial-medial junction of aortic wall. • MMP-8 and MMP-9 are increased in the site of aortic rupture where they degrade collagen, weakening the aortic wall. • MMP-1, -2, and TIMP-1 are associated with AA expansion and aortic shear stress in the AA wall. • Circulating levels of NGAL are increased in patients with AA. • ADAM10 and ADAM17 are expressed in the oldest layer of intraluminal thrombus and degrade AA collagen, favoring AA expansion. • ADAMTS1 and -4 degrade versican in the AA wall, leading to neo-intima thickening. They determine pro-inflammatory alterations on the arterial wall.
**MPs and Chronic Kidney Disease**	• Circulating MMP-2, -7, -8, -9, and NGAL levels are increased in Chronic Kidney Disease patients • MMP-9 is directly related to albuminuria levels. • MMP-2 and -9 have been found to be increased in several CKD etiologies and promote tubular damage, renal inflammation, and fibrosis. • ADAM17 cleaves ACE2, leading to an imbalance of the RAAS system; it degrades protein C receptor on the endothelium, blocking the anti-inflammatory properties of protein C. • ADAM10 degrades CADM1 and E-cadherin, determining alterations in cell-to-cell adhesions. • ADAM10 and ADAM17 degrade CXCL-16, damaging podocyte and tubular cells in the kidney. • ADAMTS13 deficiency has been associated with thrombotic thrombocytopenic purpura, glomerular, and tubular damage.

## Data Availability

No new data were created or analyzed in this study. Data sharing is not applicable to this article.
